# Bioprinting three-dimensional cell-laden tissue constructs with controllable degradation

**DOI:** 10.1038/srep24474

**Published:** 2016-04-19

**Authors:** Zhengjie Wu, Xin Su, Yuanyuan Xu, Bin Kong, Wei Sun, Shengli Mi

**Affiliations:** 1 Biomanufacturing Engineering Laboratory, Graduate School at Shenzhen, Tsinghua University, Shenzhen, P.R. China; 2 Department of Mechanical Engineering and Mechanics, Tsinghua University, Beijing, China; 3 Department of Mechanical Engineering, Drexel University, Philadelphia, PA, USA

## Abstract

Alginate hydrogel is a popular biologically inert material that is widely used in 3D bioprinting, especially in extrusion-based printing. However, the printed cells in this hydrogel could not degrade the surrounding alginate gel matrix, causing them to remain in a poorly proliferating and non-differentiating state. Here, we report a novel study of the 3D printing of human corneal epithelial cells (HCECs)/collagen/gelatin/alginate hydrogel incubated with a medium containing sodium citrate to obtain degradation-controllable cell-laden tissue constructs. The 3D-printed hydrogel network with interconnected channels and a macroporous structure was stable and achieved high cell viability (over 90%). By altering the mole ratio of sodium citrate/sodium alginate, the degradation time of the bioprinting constructs can be controlled. Cell proliferation and specific marker protein expression results also revealed that with the help of sodium citrate degradation, the printed HCECs showed a higher proliferation rate and greater cytokeratin 3(CK3) expression, indicating that this newly developed method may help to improve the alginate bioink system for the application of 3D bioprinting in tissue engineering.

The technology to fabricate three-dimensional (3D) engineered tissue analogue structures, called 3D printing^1^, would enable researchers and clinicians to tackle the current shortage of tissues and organs needed for transplants and provide platforms for drug testing and studying tissue morphogenesis^2^. There are two different approaches using 3D printing technology in tissue engineering^3^,^4^,^5^,^6^,^7^,^8^,^9^. The first approach is used to create acellular 3D scaffolds and molds, which must be seeded with cells after fabrication^3^,^4^,^5^,^6^; the second approach is used to build tissue constructs by directly depositing cells or cell aggregates, a process known as bioprinting^7^,^8^,^9^. A crucial aspect of bioprinting is that the bioink must have printability and biocompatibility because it requires the dispensing of cell-containing media^1^,^10^. The need to operate in an aqueous or aqueous gel environment limits the choice of materials, a situation cited as a significant inhibitor to the growth of bioprinting^11^. In extrusion-based printing, hydrogels are solidified through either thermal processes or post-print cross-linking and are used for the printing of cells to produce diverse tissues, ranging from the liver to bone, using materials such as alginate/gelatin chitosan/gelatin, gelatin/fibrinogen and gelatin methacrylate^12^,^13^,^14^,^15^,^16^. The alginate material system (such as alginate/gelatin) is the most popular material system in use, although it uses biologically inert material that meets the osmolar requirements of the cells, maintains their viability and hardens simply by brief exposure to calcium chloride^17^,^18^,^19^,^20^. However, there are some concerns over the outcomes of alginate studies. Derby noted that alginate systems are clearly useful for technology development purposes but are unlikely to have any long-term role because of the poor cellular adhesion that has been observed^21^. Pati *et al*. analysed the drawbacks of alginate gels and concluded that cells cannot degrade the surrounding alginate gel matrix; thus, they remain located specifically in their original deposited position during the entire culture period, limiting their capacity to proliferate and differentiate^11^. Thus, although there were some successful reports concerning the use of alginate gels to bioprint cell-printed structures, the slow and uncontrollable degradation rates of the bioprinted constructs, which induce minimal cell-proliferation and inferior cell-differentiation, are the foremost concerns.

It was already known that, after cross-linking with calcium ions, the slow degradation rate of alginate is due to the low level of released calcium ions^22^. Sodium citrate, whose citrate ion can chelate to calcium ions and form calcium citrate complexes, was proven to be an effective method to dissolve alginate hydrogels that had cross-linked with calcium chloride^23^,^24^,^25^. Encapsulating alginate beads can be completely dissolved by treating them with 55 mM sodium citrate for 20 min^23^,^24^, and the procedure of alginate sacrificing was not harmful to the cells embedded in the alginate gel^25^. Thus, sodium citrate may be a useful way to increase or even control the degradation of the bioprinted alginate constructs. Such changed matrix properties (degradation) may provide a more suitable environment in which cells can be printed and retain their capacity to proliferate and express specific marker proteins.

Moreover, although collagen has been used in various tissue-engineering applications for skin, bone and cartilage because of its good biocompatibility and low antigenicity^26^, its application for 3D-bioprinting still has limitations. In previous studies, collagen was most often used in inkjet bioprinting, which prints materials with low viscosity^27^ but rarely in extrusion bioprinting. Extrusion bioprinting needs the bioink to be self-supporting for layer-by-layer fabrication; additionally, the bioink must be temperature-sensitive and have the ability to gel rapidly on the printing substrate with high viscosity for printing definition^16^. Thus, in extrusion bioprinting, gelatin rather than collagen, has often been used as a bioink because its property fits the requirement mentioned above. However, to better mimic tissue-specific extracellular matrixes (ECM), collagen, which is the most abundant component of ECM, is used as printing materials in extrusion bioprinting.

Human corneal epithelial cells (HCECs), which are native resident cells of the cornea connected to form a dense plasma membrane^28^,^29^, were chosen to print in this matrix. HCECs were applied for seeding onto some substrates, such as amniotic membranes, collagen gel and polycarbonate membrane, to generate a bioengineered corneal epithelium^30^,^31^,^32^,^33^,^34^,^35^. However, thus far, there are no reports on the 3D printing of such cells. Considering that the advances in 3D bioprinting have enabled the direct assembly of cells and ECM to form cellular models for 3D *in vitro* biology, we used the 3D hydrogel-based cell-laden technique to print HCECs and fabricated a 3D-engineered corneal epithelium.

Therefore, in this study, we report the 3D printing of HCECs into cell-laden constructs using an extrusion-based 3D cell-printing machine that is considered to be suitable for rapid prototyping and quick fabrication of 3D organic materials^36^,^37^. Briefly, to make an environment capable of 3D cell printing, the low-temperature forming room, refrigeration function, cleaning function and sterile function need to be integrated as shown in Fig. 1. A nozzle system was developed that had a wide range of temperatures (−5 °C–150 °C) and precise temperature control (±0.1°), so that the material can be not only heated but also cooled. To obtain a construct with a good condition, we optimised the process parameters, temperature and extrusion speed that were presented in previous papers^36^,^37^. To better mimic corneal-specific ECM, the additional amount of collagen that can be added into the alginate/gelatin system to print and form a stable 3D hydrogel macroporous network was determined in this study. Immediately after printing, the viabilities of the printed cells were evaluated. To accelerate the degradation rate of the alginate/gelatin/collagen gel, we tested whether the addition of different amounts of sodium citrate could result in different degrees of degradation of the gel and determined the relation curve between them. Finally, the effect of sodium citrate on proliferation and other key biological functions, such as specific marker protein synthesis, on HCECs printed within the constructs was evaluated.

## Materials and methods

### Cell culture

Human corneal epithelial cells (HCECs)^30^,^31^,^32^,^33^,^34^,^35^,^38^ were obtained from the RIKEN Biosource Center (Tsukuba, Japan). Cells were maintained in fresh medium comprising Dulbecco’s modified Eagle’s medium (DMEM)-F12 (50:50, v/v; Invitrogen) supplemented with 10% foetal bovine serum (FBS; Hyclone), bovine insulin (5 μg/ml; Thermo Fisher), recombinant human epidermal growth factor (10 ng/ml; Thermo Fisher) and 100 U/ml penicillin and 100 μg/ml streptomycin (Invitrogen) in a CO_2_ incubator at 37 °C and with 5% CO_2_. The cells were subcultured by trypsin (0.25%; Invitrogen) and dissociated at approximately 80% confluence. The culture media were changed every 2–3 days.

### Material preparation

Gelatin powder (type A; Sigma) and sodium alginate powder (Sigma) were dissolved in phosphate-buffered saline as described in previous studies^36^,^37^. A gelatin/alginate solution was added to a neutralised rat-tail type I collagen (First link) solution with final concentrations of 1% (w/v) alginate, 10% (w/v) gelatin and 0.513, 0.615, 0.82, and 1.025 mg/ml collagen prior to the printing process. HCECs were collected by centrifugation at 1500 rpm for 3 min. The cells were dissociated into single cells and then gently mixed with the gelatin-alginate-collagen solution to reach a final concentration of 10^6^ cells/ml.

### 3D bioprinting and culture of cell-laden constructs

Using the 3D cell printer developed by our group, we fabricated a macroporous construct in a layer-by-layer fashion with the designed size of 30 × 30 mm in cross-section and 0.8 mm (eight layers) in thickness. The cell-laden solution was loaded into a 1-ml syringe, which was set in the nozzle. As previously described, the nozzle system was precisely controlled by the temperature controlling system, and the solution was held at 37 °C. The constructs were immersed in a 3% calcium chloride solution to chemically cross-link sodium alginate after printing. After cross-linking for 3 min, the constructs were gently washed 2 or 3 times and cultured at 37 °C and 5% CO_2_. Each construct was cultured in a 100-mm petri dish. To each dish, 8 ml of culture medium was added.

### Degradation rate

Before being cultured, a certain amount of sodium citrate solution with 55 mM original concentration was added to the petri dish to analyse the influence of the sodium citrate on the degradation rate of the construct. We changed the total amount of the sodium citrate by altering the mole ratio of sodium citrate/sodium alginate (C/A) to determine whether the change influenced the degradation of the construct.

### Live/dead staining

A two-colour fluorescence assay (LIVE/DEAD Assay; Molecular Probes, consisting of calcein as a marker of viable cells and ethidium homodimer as a marker of dead cells; Invitrogen) was employed to determine the cell viability in the constructs. Each sample was washed in PBS 3 times before staining. The samples were stained for 15 min in the dark and washed 3 times in PBS after staining. A confocal microscope (Xcellence; Olympus) was used for image acquisition. Three different fields were counted for each sample. Cell viability was calculated as (number of green stained cells/number of total cells) ×100%.

### Cell proliferation assay

The Cell Counting Kit-8 (CCK-8; DOJINDO) was used to analyse cell proliferation in the collagen/gelatin/alginate materials in either the absence or presence of sodium citrate in culture solutions according to the manufacturer’s instructions. Briefly, cells/hydrogel constructs with or without sodium citrate were washed with PBS three times. Next, 0.1 ml of CCK-8 and 1 ml of culture medium were added to each culture dish and incubated in the dark for 2 h at 37 °C. After incubation, 0.5 ml of the culture medium was transferred to a 96-well plate, and the optical density (OD) at 450 nm was immediately read. The 3D constructs without cells were treated the same way, and the CCK-8 medium was used as the blank control. Three samples were tested for each group.

### Immunofluorescence study

The 3D cell-laden constructs were fixed with a solution of 4% paraformaldehyde at room temperature for 20 min after 1, 3, and 5 days of culturing in a medium with 66.7% sodium citrate (C/A, mole ratio, %) and were subsequently permeabilised in 0.1% Triton X-100 for 30 min and incubated with 1% (w/v) bovine serum albumin (Sigma) for 30 min to block non-specific binding. The constructs were then incubated for 3 h at 37 °C with the primary antibody against cytokeratin 3 (CK3) (1:50; Abcam) and then incubated for 1 h at 37 °C in FITC-labelled secondary antibody (1:200; Abcam). Between each solution addition, the constructs were gently washed with PBS 3 times. Finally, the samples were co-stained with DAPI (Thermo Fisher) and observed by fluorescence microscopy (Xcellence; Olympus).

### Statistical analysis

All results were presented as the mean ± standard deviation (SD). Statistical analysis was performed using one-way ANOVA with a Bonferroni post-hoc test to determine the degree of significance. Statistical significance was defined as *p < 0.01, **p < 0.005.

## Results

### 3D printing of cell-laden constructs using gelatin-alginate-collagen

**Figure 1:**
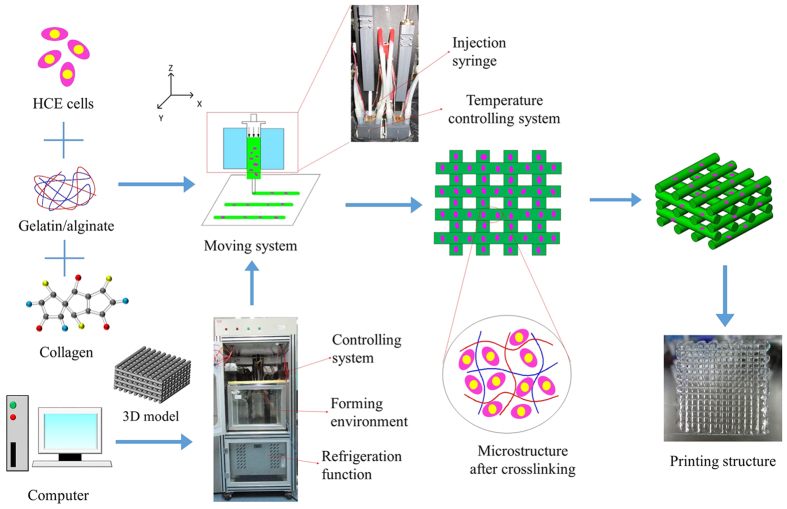
Schematic illustration of the 3D bioprinting process and optical images of the printing setup and printing constructs.

The printing of cell-laden constructs consisted of several steps; the printing setup, procedure and printing constructs are schematically summarised in Fig. 1.

**Figure 2:**
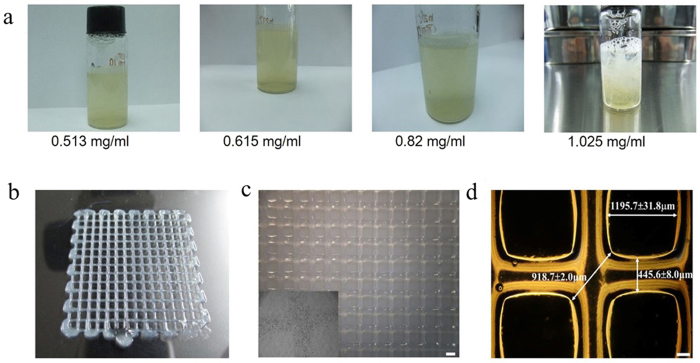
Bioprinting of HCECs in gelatin/alginate/collagen. (**a**) The different concentrations of collagen added to the gelatin/alginate bioprinting materials. (**b**) Top view of a 3D HCECs /hydrogel construct demonstrating the porous nature of the finalised scaffold. (**c,d**) The overall size images of the 3D constructs, including the pore size, thread diameter and max pore distance (c: scale bar, 1 mm; d: scale bar, 200 μm).

**Figure 3:**
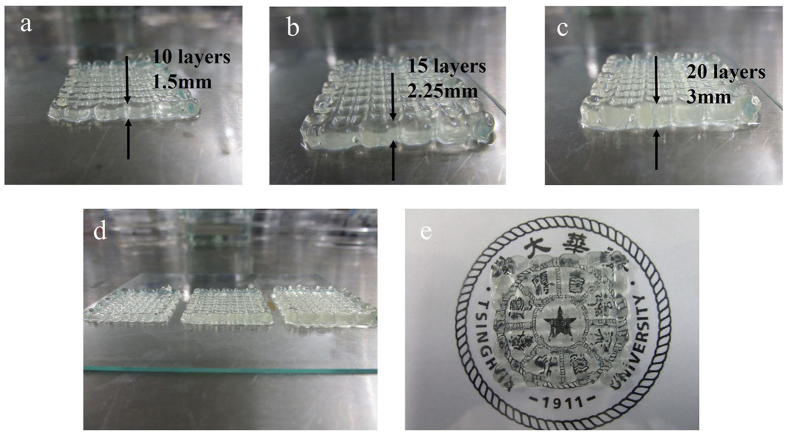
The interconnectivity of the different layers of scaffolds in the Z-direction. (**a**) Ten layers with 1.5-mm thickness, (**b**) 15 layers with 2.25-mm thickness, (**c**) 20 layers with 3-mm thickness, (**d**) scaffolds with different layers and thicknesses, (**e**) general light transmission of the scaffolds.

To better mimic corneal-specific ECM, collagen was added to the gelatin/alginate printing materials. The largest concentration of collagen that can be added to the gelatin/alginate materials to be printed and form a stable 3D hydrogel network was determined. As shown in Fig. 2a, we added four amounts of collagen to 10% gelatin/1% alginate materials and found that adding collagen concentrations lower than 0.82 mg/ml resulted in complete homogeneity of gelatin/alginate/collagen. Otherwise, the cell-laden solution appeared stratified, indicating that, in this case, collagen could not totally dissolve in the gelatin/alginate solution, undoubtedly reducing the precision of the printing scaffolds. Thus, we chose 0.82 mg/ml collagen for the subsequent experiments. Figure 2(b,c) shows the 3D-printed HCECs/gelatin/alginate/collagen constructs, which had a clear and stable structure with interconnected channels and macroporous networks. The fibres of the 3D printing constructs were uniform and smooth with a mean thread diameter of 445.6 ± 8.0 μm (Fig. 2 (d)). The interconnectivity of the different layers of scaffolds in the Z-direction is shown in Fig. 3. The thickness of the scaffolds can be precisely controlled by regulating the thickness of one layer or printing different layers (shown in Fig. 3a–c). The printing bioink (collagen/gelatin/alginate materials) has suitable mechanical properties to self-support for layer-by-layer fabrication (shown in Fig. 3d) and is suitable to be used in extrusion bioprinting. Moreover, as shown in Fig. 3e, the school logo image under the scaffold is clearly visible, and the result of light transmittance under the 630-nm wavelength of the scaffold is 62.2 ± 8.4%, indicating that the optical characteristics of the hydrogel construct is good and can be used as engineered corneal epithelium.

### Viability of printing constructs

**Figure 4:**
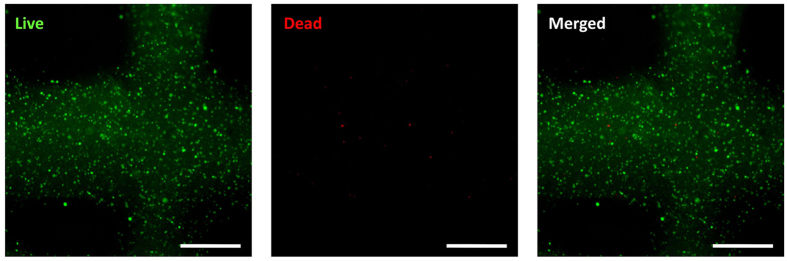
Cell viability after printing by live/dead staining (scale bar, 500 μm).

To print the cell-laden gelatin/alginate/collagen gel, one important criterion is that the material should come out of the nozzle with minimum applied shear force. Otherwise, the applied shear force may damage the cells and reduce the cell viability in the printed constructs^1^,^11^. We checked the cell viability using the live/dead assay that can be imaged in the printed construct after 3D printing. As shown in Fig. 4, there was minimal cell death caused by the printing process, and the viability of HCECs in the printed construct was found to be 94.6 ± 2.5%. Thus, the printing cell-laden materials and parameters used in this experiment did not have any harmful effect on the encapsulated cells and can support cell viability without disruption of the constructs. Although the long-term stability of the constructs during the culture time in the medium is a requirement for potential applications in 3D tissue regeneration, alginate’s nondegradable property also negatively affects bioprinted cell behaviour and tissue formation^16^.

### Effect of sodium citrate on the degradation of gelatin/alginate/collagen hydrogels, proliferation of HCECs and morphological change within the constructs

**Figure 5:**
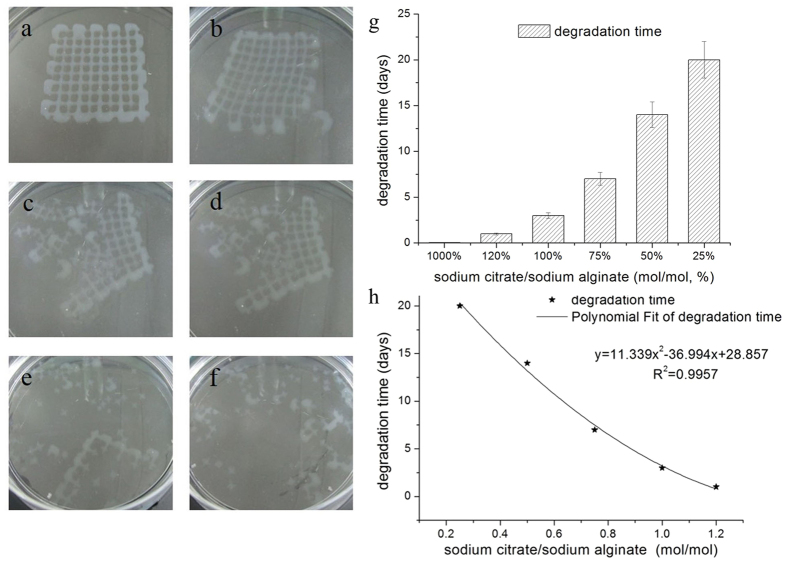
Images of the degradation process of the scaffolds set in the sodium citrate solution, from (**a**) 0 min, (**b**) 10 min, (**c**) 20 min, (**d**) 30 min, (**e**) 40 min, (**f**) 50 min, when the C/A is 1000% (mol/mol, %). (**g**) The relation of the total degradation time of the scaffolds to the mole ratio of C/A. (**h**) The relation curve between the degradation time of the printed constructs and amount of sodium citrate added to the culture medium.

Because of the drawback that cells cannot degrade the surrounding alginate gel matrix, sodium citrate was used to accelerate the degradation of alginate hydrogels. Fig. 5(a–f) shows the degradation process of the scaffolds after incubating with PBS containing sodium citrate from 0 min to 50 min. In addition, by changing the mole ratio of the C/A, the degradation time of alginate hydrogels can be controlled. As Fig. 5g shows, if the C/A (mol/mol) is higher than 1, the alginate hydrogel would completely degrade in less than 3 days. If the C/A is less than 0.5, the total degradation time would be more than 2 weeks, and the degradation time dramatically increases as the C/A decreases by even a small amount. This finding indicates that, by using this method, 3D bioprinting of degradation-controllable cell-laden tissue constructs can be successfully fabricated. Additionally, by applying the least squares method to the quadratic fitting according to Fig. 5g, the relation curve between the degradation time and mole ratio of the C/A was obtained, which is y = 11.339x^2^ − 36.994*x* + 28.857, *R*^2^ = 0.9957 (y is the degradation time, x is the mole ratio of the C/A), as shown in Fig. 5h. This formula is applicable when the amount of sodium citrate is similar to or less than the amount of sodium alginate in the system. Otherwise, the constructs would degrade too quickly to be controllable; for example, they degrade completely after 1 hour when the C/A is 1000%, as shown in Fig. 5(a–f).

**Figure 6:**
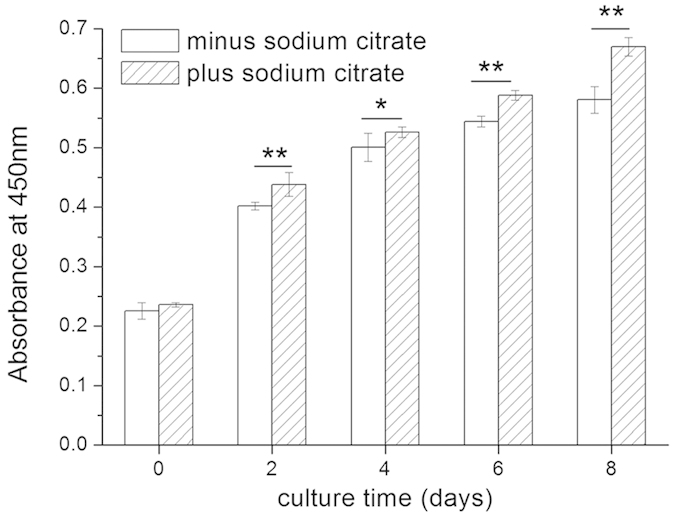
Proliferation of HCECs within the printed constructs incubated with fresh medium containing no (minus sodium citrate) or 66.7% sodium citrate (plus sodium citrate, mole ratio of C/A), as measured by CCK-8. Data represent means ± SD (*p < 0.01, **p < 0.005).

**Figure 7:**
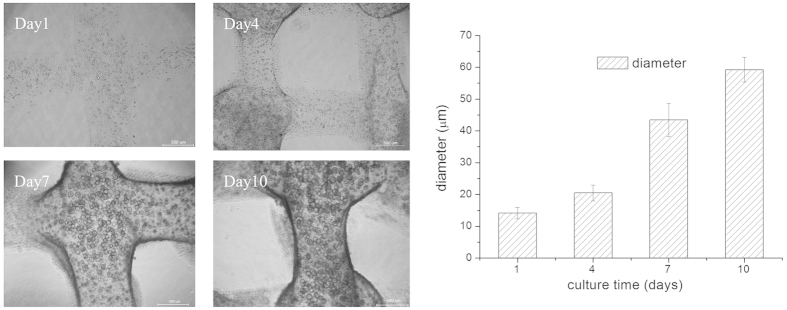
Morphological change in the cell aggregates within the printed constructs incubated with fresh medium containing 66.7% sodium citrate (C/A, mole ratio, %) during different culturing times (scale bar, 500 μm).

Moreover, the influence of sodium citrate on the proliferation of HCECs within the printed constructs was measured using CCK-8 (Fig. 6). It was found that, if the cell density was assessed immediately after measuring CCK-8, no difference in the OD was observed between the assays without (0.226) or with (0.236) sodium citrate. However, if sodium citrate was added, the cells showed faster proliferation from day 2 to day 8; on day 8, the OD values were 0.670 ± 0.015 (with sodium citrate) and 0.581 ± 0.021 (without sodium citrate). The former increased 15.4% more than the latter. The morphological changes in the 3D hydrogel during different culture times are shown in Fig. 7. The printed 3D gel constructs were incubated with fresh medium containing 66.7% (C/A, mole ratio, %) sodium citrate. From day 1 to day 10 of the *in vitro* experiments (Fig. 7), HCECs aggregated and formed clusters, which had a round-shape morphology. The number and diameter of these clusters grew with increasing culture time, which can be clearly shown in Fig. 7. The diameter of the clusters was increased from 14.15 ± 1.77 μm at day 1 to 59.22 ± 3.881 μm at day 10, which was measured using Image Pro Plus (IPP) software.

### Effect of sodium citrate on specific marker protein expression of HCECs within constructs

**Figure 8:**
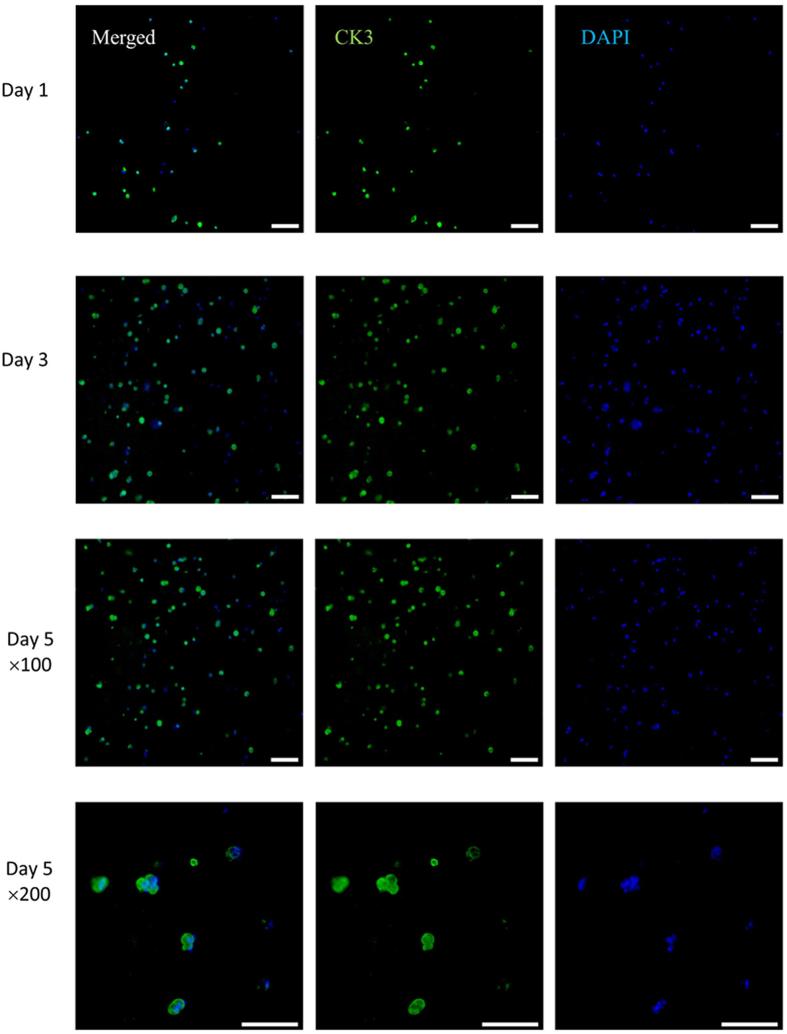
Specific marker protein expression of printed cells within constructs incubated with medium containing 66.7% sodium citrate (C/A, mole ratio, %). Micrographs show fluorescent staining of CK3 (green) and nuclei (blue) of HCECs within the constructs after culturing for 1, 3, and 5 days (scale bar, 100 μm). (For the interpretation of the references to colour in this figure legend, the reader is referred to the web version of this article.)

Corneal epithelial-specific cytokeratin 3 (CK3) is often used as a unique marker of HCECs^39^. Using sodium citrate to accelerate degradation, its expression in HCECs was achieved by immunofluorescence. After 1, 3, and 5 days of culturing in the medium containing 66.7% sodium citrate (C/A, mole ratio, %), the localization and expression of CK3 in HCECs were investigated by confocal microscopy. As Fig. 8 shows, HCECs incubated with sodium citrate proliferated during the culture time. Compared with day 1, the number of cells obviously increased at day 3 or day 5, indicating that the changed matrix property (degradation of the scaffold) may cause a higher cell proliferation, which could be the reason for the increased OD values in the CCK-8 experiment. Additionally, Fig. 8 presents largely synthesised CK3 protein by HCECs printed within the constructs, demonstrating that, using sodium citrate to accelerate the degradation process of the alginate hydrogel system, the cells can not only survive in the hydrogel but also proliferate and synthesise proteins normally. Furthermore, after 3 or 5 days of culturing, it was found that some of the cells formed two- or three-cell aggregates; this was demonstrated more clearly at greater magnification (×200).

## Discussion

Because collagen is not temperature-sensitive and has low viscosity, it is difficult to gel rapidly on the printing substrate and fabricate a scaffold with a clear and stable structure by extrusion bioprinting^16^. In previous studies, collagen was most often used in inkjet bioprinting^27^ but rarely as bioink in extrusion bioprinting. For our research, collagen was successfully printed using extrusion bioprinting technology by adding it to a gelatin/alginate system, which is widely used in extrusion bioprinting^36^,^37^,^40^,^41^. We found that, by adding 0.82 mg/ml of collagen to gelatin/alginate, the solution could achieve complete homogeneity and be suitable for extrusion bioprinting, which can fabricate a stable 3D hydrogel macroporous network. Otherwise, the solution appeared stratified and could not be used for extrusion bioprinting. The incorporation of collagen as a bioink for bioprinting not only better mimics tissue-specific ECM but also results in a more fibrous structure. Rutz *et al*. noted that the fibrous structure allowed cells to recognise the construct more readily and enhanced the robustness of the construct^16^.

Although the collagen/gelatin/alginate material system had been proven to offer many advantages in this paper, such as bioprinting with high resolution, higher cell viability at 94.6 ± 2.5% and good mechanical properties (self-supporting for layer-by-layer fabrication), its limited ability to tune degradation properties, which is not beneficial to cell proliferation and differentiation, remains a significant inhibitor to the application of this collagen/gelatin/alginate material system. Recently, some new material systems have been investigated to replace the alginate system. Pati, Cho, *et al*. reported the use of a decellularised extracellular matrix (dECM) to bioprint cell-laden constructs with an optimised microenvironment conducive to the growth of 3D structured tissue^11^,^42^. Although dECM bioink has suitable chemical composition to support cell growth and maintenance, poor mechanical properties that are not self-supporting for layer-by-layer fabrication and the lack of tailored microgeometry^22^ make them require another polymeric framework (like PCL framework) for mechanical support^11^,^42^. These polymeric frameworks are usually hard to dissolve or remove. Das printed a 3D construct with silk fibroin-gelatin bioink and used enzymatic or physical cross-linking to cross-link it^43^. However, with either enzymatic cross-linking by mushroom tyrosinase or physical cross-linking via sonication, the procedure was harmful to the cells embedded in the gel. Moreover, the cross-linked construct still showed a slow degradation rate and could not tune degradation properties. For our study, we tried to solve the problem with the lack of degradation of the alginate gel system by adding sodium citrate and controlling the degradation time of the printed constructs by altering the amount of sodium citrate that was added. Interestingly, the results not only proved that it is practical for accelerating and changing the degradation time of constructs by altering the mole ratio of C/A in the system but also indicated that, with the degrading effect of sodium citrate, the printed cells in the 3D hydrogels grew faster, had a much better capacity to proliferate and expressed greater specific marker proteins.

Furthermore, according to Fig. 5g, the relation curve between the degradation time and mole ratio of the C/A was obtained by applying the least squares method to the quadratic fitting, as shown in Fig. 5h. This formula is applicable when the amount of sodium citrate is similar to or less than the amount of sodium alginate in the system. Certainly, the degradation time is also affected by other factors, such as the printing shape, fibre spacing, and volume of the constructs. Although the formula we obtained may only be applicable for a situation using the specific printing shape, fibre spacing and volume of the constructs presented in this study, we provided a practical method to control the degradation time of printed constructs using the alginate system and cross-linking with Ca^2+^ ions.

Applying the CCK-8 kit to analyse cell proliferation, the OD values of the reagents are in a direct ratio to the number of living cells. Several studies have noted that, due to the lack of bioactive molecules in alginate, the rate of cell proliferation was extremely slow^11^,^21^,^43^,^44^. Adding collagen to an alginate hydrogel system obviously can improve the biomimetic property of the bioink and is good for the proliferation of printed cells. Incorporation of collagen resulted in a more fibrous structure that cells may adhere to and, therefore, grow much efficiently^16^. However, as the culture time increased, cells in the constructs showed a lack of degradation of the alginate hydrogel system, and their proliferation rate was found to be much slower after culturing for 2 days. Incubating with sodium citrate to accelerate the degradation process of the alginate hydrogel system is helpful for the growth and proliferation of the printed cells; this finding has been proven with the increased OD values in the CCK-8 result and increased cell numbers in the immunofluorescence staining results.

Cell-specific protein expression was used for cell definition and activity analyses. CK3 is specifically expressed in HCECs and is often recognised as a marker of HCECs^39^. The amounts of accumulated CK3-staining cells increased from day 1 to day 5 of culture, indicating that the printed HCECs can normally proliferate, synthesise and strongly express proteins in the hydrogel constructs. Additionally, it was found that HCECs aggregated and formed clusters with a round-shape morphology inside the collagen/gelatin/alginate hydrogel. These clusters grew with increasing culture time, which is shown in Fig. 7. The formation of spherical aggregates has been reported by Loessner, Zhao, Ouyang *et al*. for cells in a 3D hydrogel structure^36^,^37^,^45^,^46^,^47^. Hunt concluded that a spherical shape is the typical cell (or cell clusters) morphology inside alginate hydrogels, and no cell-material contact or cell migration out of the clusters could be noticed^48^. For our study, the results reflected that accelerating alginate degradation can obviously aid the active proliferation of cells but cannot improve cell-cell or cell-matrix interactions in bioprinting hydrogels. The process still needs to be improved by other methods. Grigore A. encapsulated cells in alginate, RGD-modified alginate and AlGel (oxidised alginate covalently cross-linked with gelatin) and compared cell behaviour^22^. It was found that cells formed spherical aggregates in both alginate and RGD-modified alginate, while cells formed cellular networks and migrated to form cell-cell contacts over large distances in AlGel^22^. Because no research has investigated whether this AlGel material system is suitable as bioink for extrusion bioprinting, it will be evaluated in bioprinting and improved to be degradation-controllable.

## Conclusion

In this study, collagen was successfully printed using extrusion bioprinting technology by adding it to a gelatin/alginate system. This matrix can better mimic tissue-specific ECM, and HCECs can achieve a high cellular viability of 94.6 ± 2.5% after printing. Meanwhile, the current work presents a useful method to solve the lack of degradation of the alginate matrix in 3D bioprinting by incubating the constructs with medium containing sodium citrate. The degradation time of the alginate hydrogels could be controlled by altering the amount of sodium citrate that was added. The results reveal that, by using this method, the printed cells in the 3D constructs grew faster, had a much better capacity to proliferate and expressed greater specific marker proteins, indicating that this work may help to improve the alginate bioink system for the application of 3D bioprinting in tissue engineering.

## Additional Information

**How to cite this article**: Wu, Z. *et al*. Bioprinting three-dimensional cell-laden tissue constructs with controllable degradation. *Sci. Rep*. **6**, 24474; doi: 10.1038/srep24474 (2016).

## Supplementary Material

Supplementary Information

Supplementary Video S1
