# Supplementary material for: Bioprinting three-dimensional cell-laden tissue constructs with controllable degradation
Source: Sci Rep. 2016 Apr 19;6:24474. doi: 10.1038/srep24474 (PMC4835808; doi:10.1038/srep24474)
Supplement: Supplementary Information [file srep24474-s1.pdf]

# Supplementary information

## Bioprinting three-dimensional cell-laden tissue constructs with controllable degradation

**Zhengjie Wu[1+], Xin Su[1+], Yuanyuan Xu[1], Bin Kong[1], Wei Sun[1,2,3*], Shengli Mi[1*]**

1.Biomanufacturing Engineering Laboratory, Graduate School at Shenzhen, Tsinghua University, Shenzhen, P.R.China

2.Department of Mechanical Engineering and Mechanics, Tsinghua University, Beijing, China

3.Department of Mechanical Engineering, Drexel University, Philadelphia, PA, USA

+These authors contributed equally to this work.

***Corresponding author:** mi.shengli@sz.tsinghua.edu.cn sunwei@drexel.edu

### Video Captions

Movie: Printing of cell-laden constructs with collagen bioink

Bioink: gelatin-alginate-collagen gel embedding HCE cells (translucent red)

Structure: interconnected channels and macroporous networks
